# The Outlook

**Published:** 1896-10

**Authors:** W. A. Yingling

**Affiliations:** Emporia, Kansas


### "THE OUTLOOK."

(Toast at Kansas State Homœopathic Society Banquet, 1896.)

By W. A. Yingling, M. D., Emporia, Kansas.

Possibly the perspectograph of Homœopathy has not been made, yet the mirror of our hindsight reflects the perspectives of the distant future. Our beloved Homœopathy has surely gained momentum during the advance of this closing century, and the velocity acquired by its own inherent force has enabled it to overcome the obstacles of ridicule and poetic puns cast in its way by allopathic jugglers. To-day the homœopathist has the respect due a professional man, if a gentleman, and stands shoulder to shoulder with his co-laborers, the allopathists, in the march toward the goal of successful practice. If with the whole world against him he has accomplished so much, and has taken possession of such vantage ground in one short century, what can he not accomplish during the opening century under such favorable auspices! To fail now would be criminal.

"Watchman, tell us of the night;
  Higher yet that star ascends.
Trav'ler, blessedness and light,
  Peace and truth, its course portends.
Watchman, will its beams, alone,
  Gild the spot that gave them birth?
Trav'ler, ages are its own;
  See, it bursts o'er all the earth."

When we adjust the microscopic lens of foreknowledge to the view of Homœopathy, the past history must largely be the gauge of reckoning. We are very apt to allow the wish to become the father of the opinion, whether it be favorable or unfavorable. The enemies of Homœopathy have long since had it dead and buried, yet to-day it occupies a broader field and a more commanding position than ever before. As friends we are apt to overlook the obstacles that will necessarily be met, especially as Homœopathy revolutionizes the opinions and practices of the past. Error and false opinions die hard from the fact that false science, or possibly it would be better to say false scientists, harbor the errors from the want of ability to calmly and disinterestedly investigate, and to accurately perceive the laws governing the intangible and higher spheres. Old notions have become fitted to the skeleton mind like old clothes to the pauper frame, and long association and familiarity cause them to be clung to with the grip of the puppy at his first root. Though we live in the boasted nineteenth century, the mental dimensions and intellectual gauge are not so greatly advanced after all. The most of that of which we boast to-day has been once before boasted of and then lost to the world by degeneration, and the balance is largely the result of chance, the happening to stumble on something waiting to be discovered. The greatest scientific knowledge seems to be the ability to know when some happy chance throws in its way a new or valuable something. That which promises much, yet antagonizes nothing, will have many advocates. That which promises more, yet is contrary to past experience and notions, and is iconoclastic of highly-esteemed opinions, will have many enemies.

Homœopathy is naturally placed in the last category. It promises more to the amelioration of man's condition and sufferings than any discovery of the past ten centuries. It is truly a revelation from Heaven through the beneficence and goodness of the Creator, yet, like all His greatest revelations, because it is antagonistic to the opinions and cultured notions of boasted higher knowledge, it is reviled, rejected, and bitterly antagonized. Although our path is yet hedged by opposition, the experience of the past warrants us in believing in the ultimate and universal triumph of the tenets promulgated by the truly scientific and philosophical mind of Samuel Hahnemann. God works in a mysterious way His wonders to perform. If God be for us—and what sane man who is able to read the lessons of history can doubt it—what matters it if the whole world—in reality only the self-assuming scientific world as contained in the interested ranks of allopathy—be against us! Truth is mighty, and will prevail by the preponderance of its own weight and merit. *Homœopathy is truth.*

Then what is the outlook? How will Homœopathy stand during the coming century? What shall the celebration of the second centenary of Homœopathy be?

I answer without reservation or doubt that the outlook for Homœopathy to-day is brighter and fairer than that of Allopathy. Allopathy is on the decline, obsolescent. The ranks are still full and the patrons are plenty, yet the mutations of the cardinal principles of the system plainly indicate the decay. The allopath of to-day is not the allopath of yesterday. The continual change in treatment points to the instability of the system. The ecstatic hailing of every new remedy as a miraculous discovery shows the felt want of something better than they now possess. The early abandonment of these will-o'-the-wisp cure-alls and sure-cures unerringly speak louder than words the utter hopelessness of allopathy. It is not yet dead, but the gangrene of a slow but sure decay is eating at its vitals, and by the next centenary of Hahnemann's art the remaining life in the body of the now prevailing system of medicine will be so low that the Thompsonian undertaker will be preparing for a job.

The outlook is bright and fair for Homœopathy. Never in its history has there been so much activity and life; never has there been such a demand for, and searching of, the writings and teachings of Hahnemann; never has there been more respect and demand for the homœopathic prescriber; never has there been more courage and assertive force on the part of the followers of Hahnemann. With the impetus now acquired, and the better facilities of both the matter and manner of applying Homœopathy, there will be a changing of places during the incoming century—the homœopathic will be acknowledged the "regular" school of medicine, the criterion of the best, the ideal; the allopathic will be remembered as the relic of the Dark Ages, while their descendants will hide their faces in shame at the mention of the teaching of the authors of to-day.

